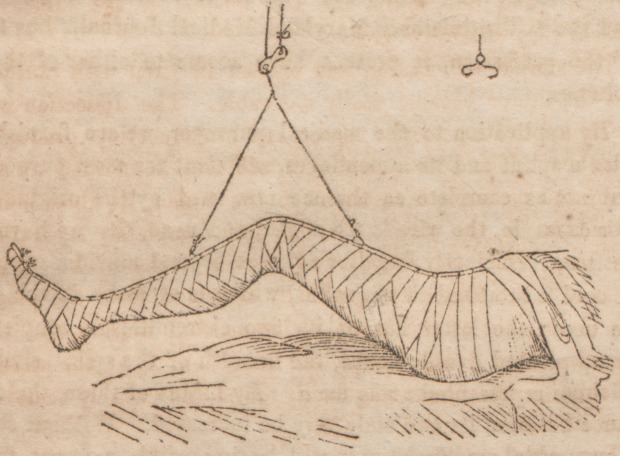# On the Application of Smith's Anterior Splint

**Published:** 1864-05

**Authors:** Russell Murdock

**Affiliations:** Assistant Surgeon P. A. C. S.


ART. III.—*On the Application of Smith's Anterior Splint.* By RUSSELL MURDOCK, Assistant Surgeon P. A. C. S.

We desire to snatch from undeserved obloquy a great boon, in our opinion, to the suffering of the army, which has unfortunately, by its too frequent misapplication, been made a very curse. We allude to the "anterior splint" used by Professor N. R. Smith, of Baltimore.

Any one, who has ever seen the proper application of the exceedingly simple and strikingly neat apparatus, has his sense of mechanical propriety shocked, as he glances through the surgical wards of many of our military hospitals, with the ungainly and uncomfortable appearances presented by the unfortunates undergoing *injudicial suspension* by their lower extremities. Let him ask any medical attendant his opinion of the treatment of fractures of the bones of the leg and thigh by means of the above splint, the response will be almost uniformly unfavorable to its use. He will be told of excoriations, ulcerations, abscesses, and deformities of all sorts; most unfortunately the information is correct.

Now "dead men," we are told, "tell no tales," but living, walking—no hobbling—limping ones do; and of this misused apparatus many bad ones are related. We have heard of one poor fellow suffering agony for weeks, yes months, in this machine, from simple fracture of the femur, resulting in angular deformity and loss of the foot itself. Immediately before applying ourselves to the present communication, we were induced, by feelings of friendship, to visit an old patient in a neighboring hospital. We found him strung up in mid air by some unaccountable connection to a piece of bent wire, with a bandage applied at intervals of some inches, not attached to the perineum or pelvis. By this neglect, the upper end of the wire, which we recognized as "Smith's splint," was at least half a foot from the body. Can he escape without "excoriation, ulceration or abscess?" These and the frequent request by friends for information upon practical points in the application of this splint, have induced us to use your well conducted and already extensively circulated sheet in endeavoring to furnish the desideratum. Articles, we are aware, have been already written on this subject, both in the "American Journal of Medical Sciences," and the "Virginia and Maryland Medical Journal," but few of the profession, at present, have access to either of these volumes.

By application to the medical purveyor, we are furnished with a splint and its appendages, sufficient for most purposes, but not as complete as the one now used by the originator. For example, the wire is too slight, and none, that we have as yet seen, sufficiently large for a person beyond medium stature. A cord is attached to the splint, which is separate in the one he uses; the latter has, at its two extremities, a couple of sharp-pointed double hooks, the breadth of the splint, similar to those represented at figure *a*. By means of these, the distance between its two ends may be increased. Without such a moveable modification, it will be impossible to treat fractures below the knee. The last censure we make on the splint has reference to a loop universally tied in the centre of the bifurcating cord attached to the splint. The purpose of balancing the splint at its centre of gravity, when applied, is entirely nullified by this loop. It prevents likewise the possibility of uniform tension along the limb when we use different degrees of obliquity in the cord.

This splint can be made either of wood or wire. For lightness and convenience in application, the wire one is that most commonly used. The first thing we do is to bandage the splint from end to end, both for neatness and compactness. In cases of compound fracture, however, when the wound is on the anterior surface, we leave fenestrated apertures over the opening or openings. The patient is placed upon his back, with both limbs free. The sound limb is taken as a criterion for proper measurement for flexures of the splint. These are three: first, immediately over the ankle joint; then at the centre of the patella; and, finally, at the bend of the groin. The foot portion should project beyond the great toe, and that for the abdomen as high as a line with the last floating rib. This is to gain an adequate hold on the splint by a spica bandage. The angles of the flexures should be of about equal degrees, viz: sixty-five degrees; differing in direction in which they look—the middle one looking upward, and the other two downwards. Two points may be mentioned at this stage, both of which are of great importance; cross pieces of wire, intended to strengthen the splint in a majority of cases, fall upon the knee or ankle joints. This can easily be remedied by knocking them out of the way prior to bandaging the splint itself. The neglect of this precaution has, in one case which came under our notice, produced ulceration into the ankle-joint. The second point has reference to the plane of the abdominal portion. All the rest of the splint has its plane, in breadth, horizontal; but here we are necessitated to adapt it to the abdominal parietes by bending it downwards and outwards. Cotton batting of sufficient thickness, or five or six layers of cotton cloth, the breadth of the splint, is placed along the whole anterior surface of the fractured limb, excepting in cases of compound fracture, with the wound in front, as above mentioned. An assistant now supports the limb at the knee, making sufficient traction *to adapt the limb*

*to the flexures made in the splint*, which, being an exact mould of the sound limb, must necessarily prevent shortening. Another supports the heel, keeping the great toe pointing upwards. No side splints are necessary, except where there is great tendency to lateral displacement. The surgeon now takes a roller bandage in his hand, applies a few interrupted pieces along the limb, pinning them on the top, over the splint, as a temporary support to the limb: one at the middle of the sole of the foot; a third below the knee, and a fourth and fifth at the lower and upper thirds of the thigh. Prior to adjusting, we have caused to be driven into the ceiling or rafter a staple, and through it we pass the loose end of the supporting cord, and then attach it in the upper hole of the block. It is hardly necessary to mention that this block is simply for convenience in elevating and depressing the limb as required under different circumstances. Sufficient cord, and some to spare, is generally furnished; what is over may be either rolled up or cut off. The unattached end, provided with a hook, should be made to hang sufficiently high to keep the heel two or three inches from the bed, hooking it on to the centre of gravity of the bifurcating cord Another requisite is, that this cord should hang immediately over the centre of the patella when we are applying the splint. The bed should, at this stage, be advanced from the wall, so as to be moved back, as far as necessary, after the application of the splint. By this means we get, in part, the extension to the limb by the obliquity of the cord thus produced. This obliquity varies in different cases; as a general rule, the higher up the bone the fracture is, the greater the obliquity required. We are governed pretty much by the facility of overcoming the muscular contraction and the comfort of the patient. In fracture of the bones of the leg, no obliquity is required.

The tendency of the cord is to become vertical, that of the limb to assume the horizontal, and the resultant force of these two forces pulls along the course of the fractured femur. We are of opinion, however, that much less extension by this means is necessary than is generally supposed; the limb is bandaged along the whole length of a rigid splint, precisely moulded to the limb, which seems to us to be a very potent means of overcoming the tendency, of the fractured bone, to override The *two* certainly, however, are adequate to prevent shortening in all cases. If there is a tendency in the patient to slip down in bed, and consequently to allow the cord to assume the vertical, we can counteract it by elevating the foot posts of the bed.

The next stage is to bandage the limb closely, not *tightly*, to the splint from toe to groin, and from thence to the pelvis, the latter effected by the spica bandage. The whole of this is placed over the cotton batting, splint and interrupted bandage; this latter is left to support the limb should the bandages become soiled. No tumefaction bandage is required. In cases of compound fracture we require the following modification in the bandage, viz: to arrest our bandage below the wound, and re-commence another above it. The space left is intended for the application of dressings—which should be changed as often as deemed necessary—and over them an independent bandage. Should this permanent bandage be soiled, or should it hurt the patient at any place, it should at once be taken off, the part examined, relieved, and then properly re-adjusted. This is a very easy matter; but a general opinion prevails that, when once applied, it should never be touched. Now we know no splint in which the test of its proper application is perfect freedom from pain except this one. We next move the bed back to produce the required obliquity. The heel should be raised not more than two inches from the bed.

Covering for the patient is effected by doubling two sheets lengthwise, stitching them as far down as the first arm of the bifurcating cord, and pinning them below. The blanket and coverlet in the same manner.

In closing, we would remark that this apparatus has been successfully used in fractures of the bones of the thigh and and leg, Potts' fracture excepted—in diseases of the hip and knee joints—in painful ulcerations and wounds—extensive abscesses where uniform compression and rest is desired—and where extension as well as rest is required, as in coxalgia, it is likewise beneficial.

Had we not taken already so much of your valuable space, we would like to give the satisfactory results of cases corresponding to the above classes treated by this instrument.

I trust that the unfavorable statistics, unjustly charged to the score of this most useful apparatus, but by rights to its misapplication, may be revised, or at least that we may have better success attending its use in the future.

## Figures and Tables

**Figure f1:**